# Diagnostic Yield of Sequencing for Prenatal Diagnosis of Fetal Structural Anomalies: An Updated Systematic Review

**DOI:** 10.1002/pd.70112

**Published:** 2026-03-02

**Authors:** Karen Mei Xian Lim, Alexander Gibbs, Elizabeth Scotchman, Graeme Smith, Lyn S. Chitty, Natalie J. Chandler

**Affiliations:** ^1^ Department of Obstetrics and Gynaecology National University Hospital Singapore Singapore; ^2^ NHS North Thames Genomic Laboratory Hub Great Ormond Street Hospital for Children NHS Foundation Trust London UK; ^3^ Genetics and Genomic Medicine UCL Great Ormond Street Institute of Child Health London UK

## Abstract

The clinical utility of sequencing in prenatal diagnosis is known, but diagnostic yield varies widely depending on clinical indication. Here we update an earlier systematic review reporting the diagnostic yield of prenatal sequencing in structurally abnormal fetuses, with particular focus on factors affecting diagnostic yield. The search strategy outlined in the previous review (2018–2022) was repeated to include reports published between October 2021 and January 2025. Combining these records with those from the earlier review, an overall incremental diagnostic yield of sequencing compared to chromosomal microarray analysis (CMA) was calculated and pooled in a meta‐analysis. Subgroup analyses were performed on selected, unselected, and exploratory cohorts, as well as individual phenotypic subgroups. 155 records were reviewed (89 from the current and 66 from the earlier publication). The overall pooled diagnostic yield of sequencing above CMA across all indications was 27% (95% CI 24%–30%, *p* < 0.0001). Subgroup analysis showed that the diagnostic yield was 40%, 20%, and 17% in selected, unselected, and exploratory subgroups, respectively. Concurrent CNV analysis of sequencing data provided nine additional diagnoses over sequential sequencing after a non‐diagnostic CMA. Prenatal sequencing provides an additional yield of 27% following a non‐diagnostic CMA across a range of fetal structural anomalies, and this increases to 40% with careful case‐selection. Sequencing continues to be a powerful diagnostic tool when performed for well‐evidenced indications.

## Summary

- What's already known about this topic?
  ◦ Exome and genome sequencing provides significant additional diagnostic yield in structurally abnormal fetuses with a non‐diagnostic chromosomal microarray analysis
- What does this study add?
  ◦ Diagnostic yields of prenatal sequencing studies performed in phenotypic subgroups demonstrate additional yield across most indications
  ◦ Copy number variant detection performed concurrently with sequencing may provide additional diagnoses

## 1 Introduction

In the last decade, sequencing has become an increasingly important diagnostic tool in elucidating the etiology of major fetal structural abnormalities identified on imaging. An earlier systematic review in 2022 showed that exome sequencing provided a diagnosis in an average of 31% of cases with a non‐diagnostic chromosomal microarray analysis (CMA) in fetuses with a structural malformation [1]. When sequencing is performed in highly‐selected indications using a multidisciplinary team (MDT) approach, diagnostic yields of up to 81% [2] have been reported. In recent years, the decreasing cost of sequencing has resulted in a commensurate increase in publications on prenatal sequencing. Additionally, the discovery of new gene‐disease associations and case‐level evidence generation may have increased diagnostic yields over time. However, despite knowledge of its utility in clinical practice, the heterogeneity of data from prenatal studies may limit the generalizability of results to guide clinical decision making [3]. The need for more specific diagnostic yields is evidenced by the publication of several phenotype‐specific systematic reviews in recent years [4, 5, 6, 7, 8, 9, 10, 11, 12, 13, 14, 15, 16, 17]. Additionally, eligibility criteria for prenatal sequencing practices have been shown to vary widely between countries [18], adding further to the heterogeneity of published data. Lastly, bioinformatic pipelines and reporting practices vary across the world [19], which can also affect the detection of diagnostic variants [20].

Here, we present an update of the 2022 systematic review by Mellis et al. [1] to obtain an overall diagnostic yield. Importantly, we explore the factors known to affect diagnostic yield, such as case selection, inclusion criteria, sequencing approaches and reporting strategies, performing subgroup analyses where appropriate.

## 2 Methods

### 2.1 Protocol and Registration

The systematic review protocol is as described by Mellis et al. [1]. The same research protocol was applied to records published between October 2021–January 2025 to cover the period after the first review. The review was registered prospectively on the PROSPERO international register of systematic reviews (reference CRD420250644200).

### 2.2 Eligibility Criteria

Records included in this review met the following criteria: (i) retrospective or prospective cohorts of 10 or more pregnancies undergoing exome sequencing (ES) or genome sequencing (GS) for diagnosis of fetal structural anomalies, (ii) chromosomal analysis via CMA or karyotyping was non‐diagnostic, (iii) testing was initiated based on the prenatal phenotype, and (iv) full text report was available in English language. Case reports, reviews, editorials, and commentaries were excluded.

### 2.3 Information Sources and Search Strategy

Electronic searches of four databases (MEDLINE, Embase, Cochrane library, and Web of Science) were conducted for records published between October 2021 and January 2025. Potentially relevant studies were also identified by manually searching reference lists of relevant studies and published reviews.

Search terms were variations on the keywords “prenatal diagnosis” and “exome sequencing analysis, DNA.” Alternative terms for “prenatal diagnosis” included “fetal,” “fetus,” “prenatal” and “antenatal.” Alternative terms for “exome sequencing analysis, DNA” included “exome sequencing,” “whole exome sequencing,” “exome,” “whole genome sequencing,” and “genome, human.” The full search strategy is detailed in the systematic review by Mellis et al. [1].

### 2.4 Study Selection

After removal of duplicates, three reviewers (A.G., L.C. and E.S.) independently screened titles and abstracts. For abstracts deemed potentially relevant, full text articles were retrieved and reviewed against the inclusion and exclusion criteria by A.G. and K.L. Any disagreement between reviewers was resolved by discussion.

### 2.5 Data Extraction

Where the information was available, the following data was extracted by one author (K.L.): study setting, sample size, inclusion criteria, sequencing approach, gestation at testing, turnaround time, number of fetuses with diagnostic variants, variants of uncertain significance (VUS), incidental findings, and whether variants were re‐analyzed. In studies where CNV analysis was performed in parallel with sequencing, aneuploidies and pathogenic CNVs which would have been detected by CMA (> 100 kb) were removed from the sample size and overall diagnostic yield. Exonic CNVs detected by sequencing as well as cases where a pathogenic CNV was detected by CMA and found to be in trans with a pathogenic single nucleotide variant (SNV) were counted as cases diagnosed by sequencing. Only pathogenic and likely pathogenic variants which explained the prenatal phenotype detected by sequencing were considered “diagnostic” and included in the overall diagnostic yield. In studies where re‐analysis of data was performed, variants that were upgraded to pathogenic following re‐analysis were not included in the overall diagnostic yield.

To determine the diagnostic yield in fetuses with defects confined to a specific phenotypic subgroup, data were extracted from two sources: studies which included fetuses with any major anomaly but provided subgroup data on the diagnostic yield in fetuses with single system anomalies, and studies which assessed diagnostic yield in a particular phenotypic group (i.e., congenital heart defects). When available, data regarding fetuses with an isolated defect confined to a specific system or phenotype were extracted. When this information was not available, the corresponding authors were contacted for more data.

To determine the role of case‐selection based on well‐evidenced criteria or expert opinion, studies were classified based on their method of case‐selection for further subgroup analysis. Accordingly, studies were classified as selected if:

- Cases were selected by MDT or expert opinion as having a high likelihood of monogenic disorder, or
- Included phenotype was already known to have high diagnostic yield based on strong evidence (e.g., short long bones suggestive of skeletal dysplasia), or
- Sequencing was offered based on national well‐evidenced criteria [18, 21, 22, 23].

Studies were classified as unselected if:

- Inclusion criteria were any major fetal anomaly that did not undergo MDT or preselection (e.g., “consecutive cases”), or
- No information was provided on selection criteria but included phenotypes which were both evidenced and not well‐evidenced
- Studies were classified as exploratory if: Inclusion criteria was a phenotype/phenotypic group not currently included in well‐evidenced national criteria (e.g., isolated thickened nuchal translucency), or
- Performed in a phenotypic subgroup (e.g., CAKUT) but included high‐ and low‐risk phenotypes (e.g., hyperechogenic kidneys but also isolated renal cysts)

If there was insufficient information regarding selection criteria, the study was classified as unclear and not included in the subgroup analysis.

### 2.6 Quality Assessment of Included Studies

The quality of the studies was assessed using a modified Standards for Reporting of Diagnostic Accuracy (STARD) checklist used in the previous review. An author (M.H.) from the original review [1] was consulted about the use of the checklist to ensure consistency. Quality assessment of the first five studies in this review was performed independently by two reviewers (E.S. and K.L.), and discrepancies were resolved by discussion. Once consensus was reached on how to apply the criteria, the remaining studies were assessed by K.L. only.

### 2.7 Data Synthesis

Our primary outcome of interest was the incremental diagnostic yield of sequencing compared with chromosomal analysis via CMA or karyotype, expressed as a risk difference. This was calculated for each study with 95% confidence intervals and pooled for all studies in our meta‐analysis, using a random‐effects model with inverse variance weighting. Studies reporting data on isolated system anomalies classified by different phenotypic groups underwent subgroup analyses to generate estimates of pooled incremental yields by phenotypic groups. Findings were displayed using forest plots. Between‐study heterogeneity was assessed visually using an *I* ^2^ statistic. Publication bias was assessed graphically using a funnel plot with Egger's test. All statistical analyses were performed in R Statistical Software (v4.5.1, meta package 8.2‐1) [24, 25].

## 3 Results

**FIGURE 1:**
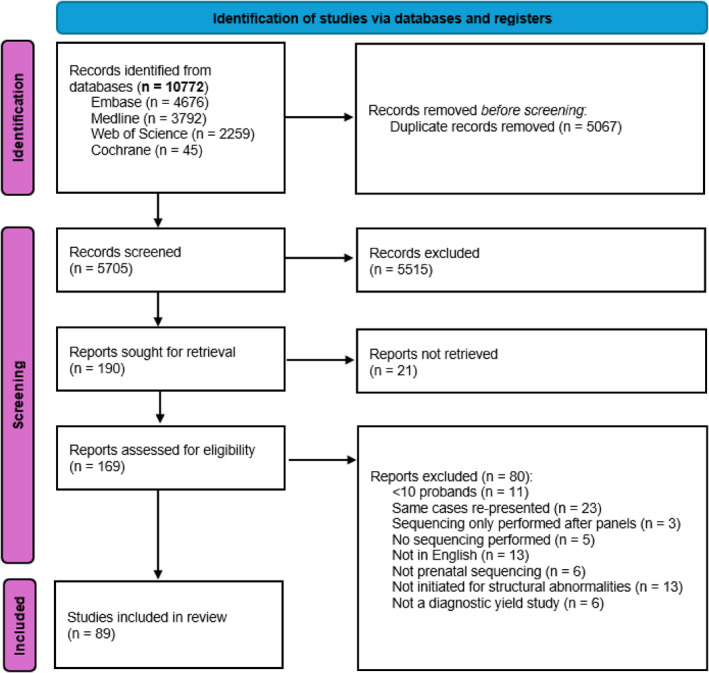
PRISMA flow diagram showing study screening and selection.

Database searches identified 5705 unique records. After screening by title and abstract and retrieving full text reports, 150 records were assessed for eligibility. Of these, 89 records were deemed eligible for inclusion in the final review (Table S1). This was combined with the 66 records included in the earlier review [1] to give a total of 155 records (Figure 1).

### 3.1 Study Characteristics and Demographics

**FIGURE 2:**
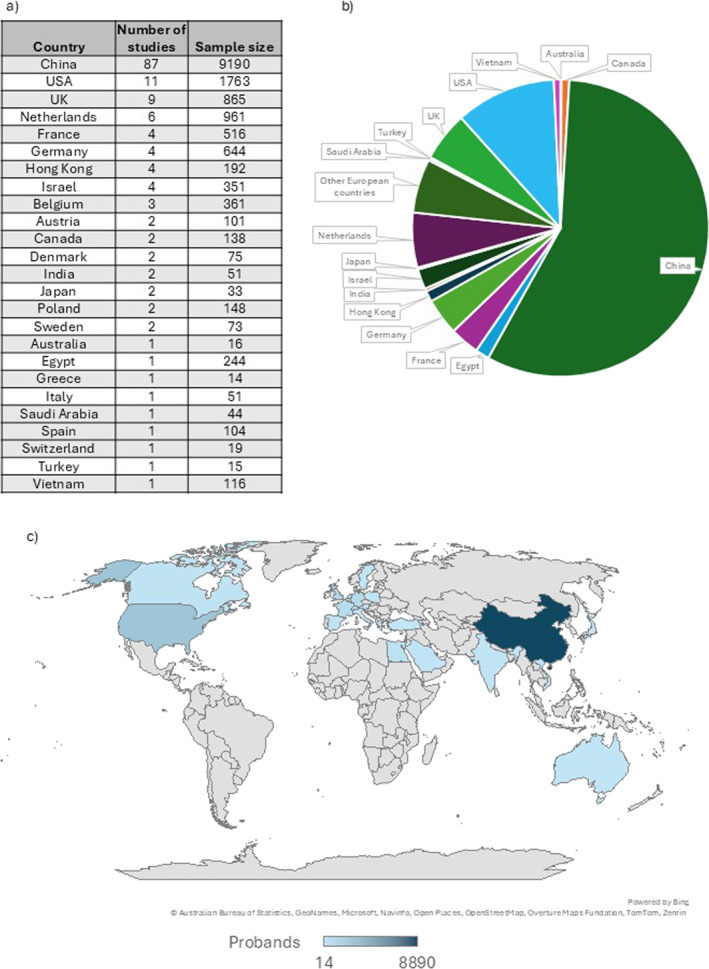
Distribution of countries where fetal sequencing studies were conducted. (a) Total number of studies and pooled sample size across all 155 included studies. (b) Number of probands sequenced from each country/region. (c) World map indicating the countries which performed sequencing and were included in this review. The shaded color of the country represents the corresponding sample size, with larger sample sizes indicated by darker shades of blue.

**FIGURE 3:**
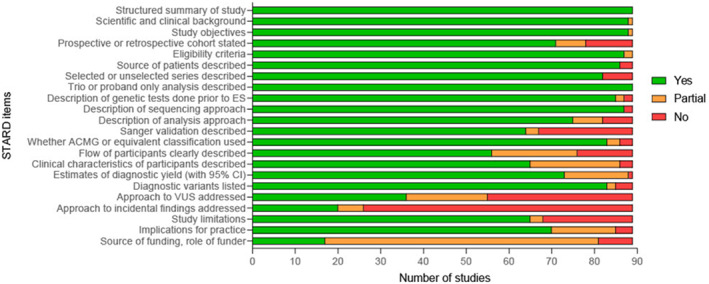
Quality assessment of 89 records obtained from the updated review using modified Standards for Reporting of Diagnostic Accuracy (STARD) criteria.

One hundred fifty‐five records were published from 25 countries (Figure 2a–c), representing 15,695 probands. The indication for sequencing was one or more major fetal anomalies detected on ultrasound in 44.5% of the studies (69/155), and for anomalies from a specific phenotypic group (i.e., central nervous system) in the remaining 86 studies (55.5%). The quality of the reports included was generally high, as assessed using modified STARD criteria represented in Figure 3.

### 3.2 Sequencing Approaches

ES was the approach used in the majority of studies (121/155, 78.1%), while GS was performed in 16 studies (10.3%). Clinical exome sequencing (CES), which we define as targeted capture of genes related to Online Mendelian Inheritance in Man (OMIM) disease genes, was performed in 19 studies (12.3%). Targeted panels were used as the only sequencing method (4/155, 2.6%) or in combination with ES (3/155, 1.9%). Virtual gene panels were applied in 34 studies on exome or genome sequencing data. This refers to restricting the tertiary analysis of sequencing data to a limited panel of genes, which differs from the earlier defined targeted panel capture. The main difference between the two lies in the ability to expand the analysis to the entire exome or genome if no significant variants were found in the virtual panel.

### 3.3 Copy Number Variant Detection

While most studies performed a chromosomal analysis via karyotype and/or CMA to exclude aneuploidy and pathogenic CNVs before performing sequencing, four studies performed ES or GS with concurrent CNV detection using CMA or low‐pass genome sequencing as the first‐line investigation [26, 27, 28, 29]. Separately, 40 studies performed CNV analysis in parallel with sequencing even after a non‐diagnostic CMA. This was done either by separate low‐pass genome sequencing or CNV‐calling from sequencing data. From the studies which performed CNV assessment in parallel as part of sequencing, three types of diagnoses were attributed to using this approach. These were the detection of small pathogenic intragenic CNVs which were smaller than the resolution of CMA, dual diagnoses of a pathogenic CNV and SNV occurring in a single patient, and compound heterozygotes for a pathogenic CNV and SNV in an autosomal recessive (AR) gene which explained the phenotype. All CNVs detected from sequencing data were confirmed using a validated method (either CMA or quantitative PCR).

### 3.4 Comparisons of Study Characteristics

Comparisons between the characteristics of studies from this and the previous review are presented in Table 1, demonstrating the differences in sequencing approaches and inclusion criteria.

**TABLE 1:** Comparisons of studies from previous review and current review.

	Mellis et al.	Current review
Period of review	January 2010–October 2021	October 2021–January 2025
Number of records for full review	66	89
Sequencing approaches		
Targeted panel only	4 (6.1%)	0
Exome sequencing^a^	57 (86.4%)	78 (87.6%)
Genome sequencing^b^	5 (7.6%)	11 (12.4%)
Selected	34 (51.5%)	32 (36.0%)
Unselected	18 (27.3%)	22 (24.7%)
Exploratory	10 (15.2%)	34 (38.3%)
Inclusion criteria		
Any major structural anomaly	35 (53.0%)	34 (38.2%)
Phenotypic subgroups	31 (47.0%)	55 (61.8%)
Central nervous system	3	14
Cardiovascular system	5	13
CAKUT	2	8
Skeletal	9	6
Hydrops	4	2
Thickened NT	4	6
Neuromuscular	2	2
Gastrointestinal	0	2
Craniofacial	2	1
Respiratory	0	1
Concurrent CNV analysis with sequencing	14 (21.2%)	30 (30%)

^a^ Includes clinical exome sequencing and studies which performed targeted panel capture for the initial stages of the study exome sequencing for the rest of the cohort.

^b^ Includes studies where either exome or genome sequencing was performed.

### 3.5 Incremental Diagnostic Yield From Sequencing

The overall pooled diagnostic yield of sequencing over chromosomal analysis via CMA/karyotype across all 155 studies was 27% (95% CI 24%–30%) (Supporting Information S1: Figure S1). The pooled diagnostic yield of sequencing from the 89 studies in the updated review was 25% (95% CI 22%–27%). Between study heterogeneity was high (*I* ^2^ = 88.4%, *p* < 0.0001), with Egger's test indicating possible publication bias (*p* = 0.002) (Supporting Information S1: Figure S3).

### 3.6 The Effect of Case Selection

The pooled diagnostic yield was 40% (95% CI 35%–44%) in selected cohorts. In studies where the cohort was unselected, the pooled diagnostic yield was 20% (95% CI 17%–24%), and 17% (95% CI 14%–20%) when done for exploratory indications (Figure 4a–c). The difference in diagnostic yield between selected and unselected cohorts was statistically significant (*p* < 0.001). Examples of phenotypes included in the exploratory cohorts were micrognathia, isolated talipes, and bowel dilatation. An example of an “exploratory” subgroup was congenital heart defects, which contained a mix of low‐risk phenotypes such as ventricular septal defects and high‐risk phenotypes like conotruncal defects. Notably, congenital cardiac defects are found on some [18, 22], but not all published national inclusion criteria for exome sequencing, reflecting a lack of consensus about its inclusion. The three studies with the highest diagnostic yields in the exploratory cohort were 61.5%, 42.9%, and 42.1% in fetuses with apparently isolated micrognathia in the first trimester [30], bilateral renal agenesis [31], and non‐compaction cardiomyopathy [32], respectively.

FIGURE 4(a) Forest plot showing individual and pooled incremental yield of prenatal sequencing over karyotype/chromosomal microarray for “selected” cohorts (*n* = 65), (b) “unselected” cohort (*n* = 40), (c) “exploratory” cohort (*n* = 45).

**Figure:**
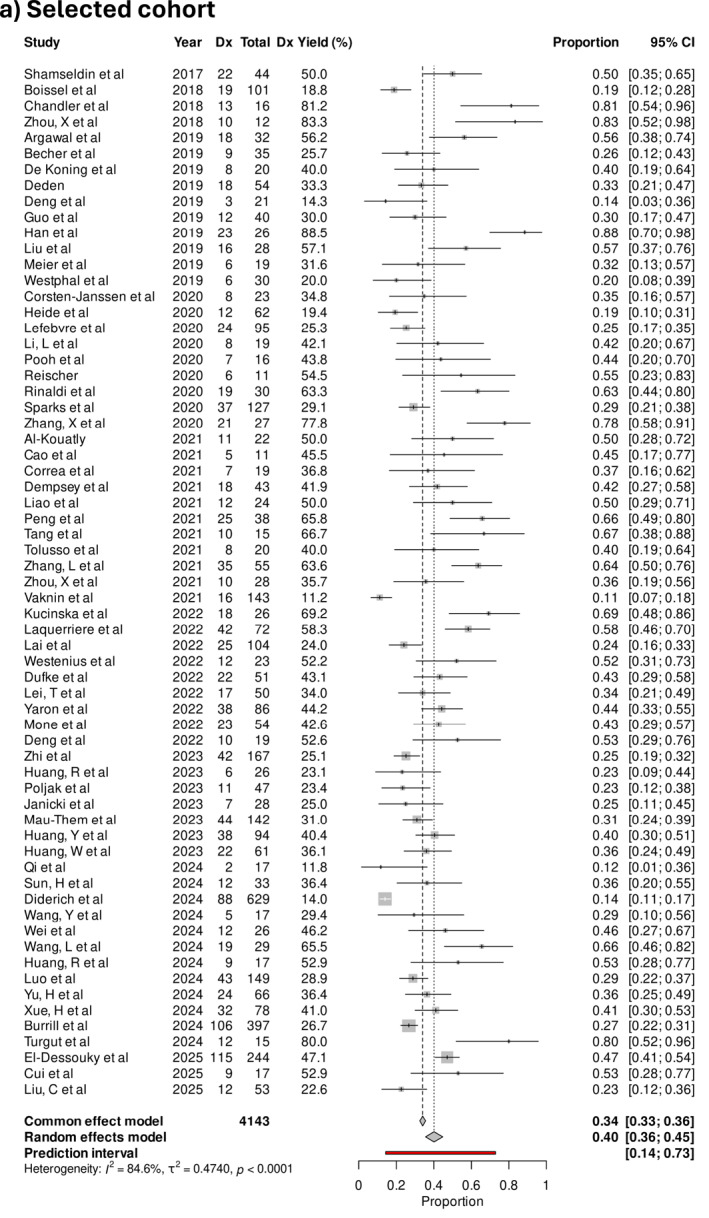

**Figure:**
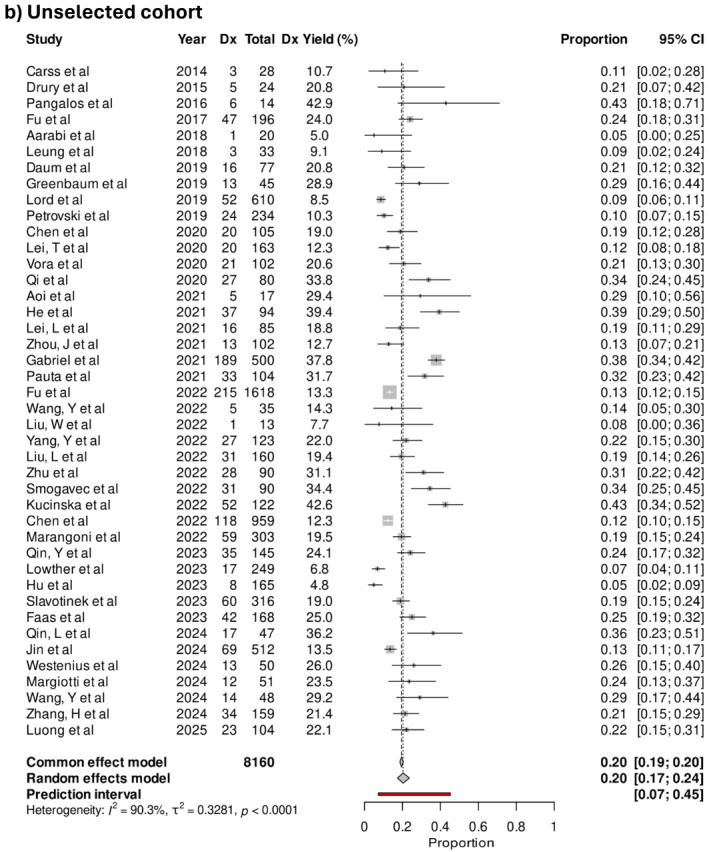

**Figure:**
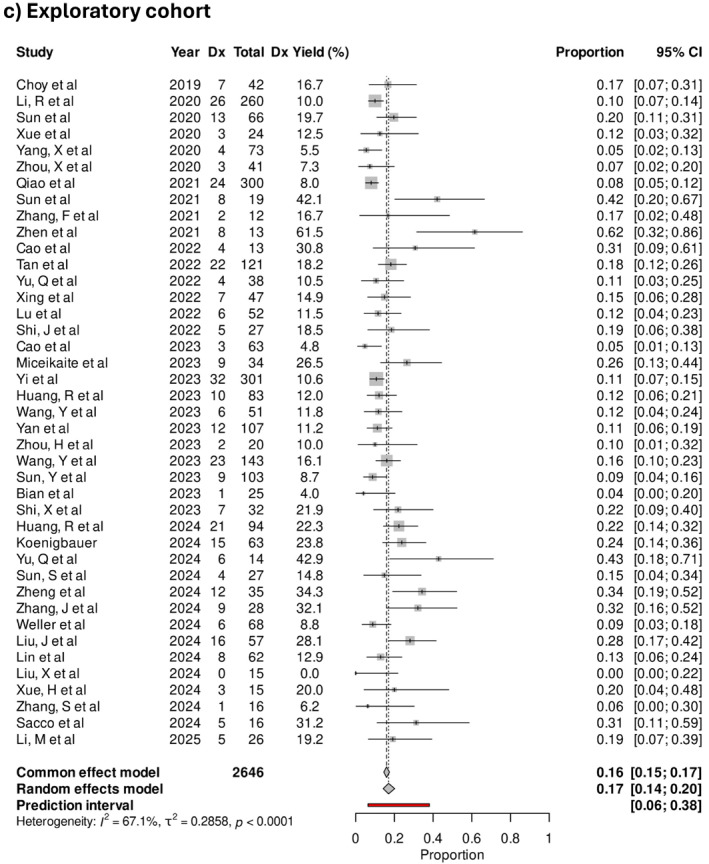

### 3.7 Variants of Uncertain Significance and Incidental Findings

One hundred thirteen studies reported variants of uncertain significance (VUS), with an average VUS rate of 14% (95% CI 12%–16%) across all studies. The average VUS rate appeared lower when sequencing was performed as a trio (13.0%) compared to when sequencing was performed in the proband only (19.0%), but this difference was not significant (*p* = 0.07). Incidental findings were described in 50 studies (32.3%). In studies that specifically reported secondary findings, defined as medically actionable secondary findings as per American College of Medical Genetics and Genomics (ACMG) policy statements [33], these were only reported in the parents but not in the fetus.

### 3.8 Reporting Methods

ACMG criteria for variant classification were used in all except eight studies. However, in studies which reportedly applied ACMG criteria to variants, several did not describe the evidence criteria applied when reporting individual diagnostic variants. Additionally, one study considered cases with a VUS in *trans* with a pathogenic variant in an AR gene consistent with the phenotype as a positive diagnosis. Ten studies performed re‐analysis of results [34, 35, 36, 37, 38, 39, 40, 41, 42, 43]. This was most commonly performed when additional phenotypes were available either during pregnancy or postnatally (seven studies). In studies which described the total number of re‐analyzed cases, 4.7%–18.8% of analyzed variants were upgraded using new phenotypic information [34, 36, 38]. However, only three studies described performing re‐analysis when a new structural malformation was found on ultrasound in later gestation, while the rest utilized postnatal information [34, 36, 39]. New gene‐disease associations resulted in four cases being upgraded to pathogenic after the initial analysis [36, 43]. However, the total number of re‐analyzed cases using this approach was not described. One study reported a laboratory policy of performing routine re‐analysis of undiagnosed cases after 1 year [43].

### 3.9 Phenotypic Subgroups

The pooled diagnostic yields of all studies and of each phenotypic subgroup are presented in Table 2 and Supporting Information S1: Figure S2. Comparisons are made with results from the earlier review by Mellis et al. [1] and other systematic reviews.

**TABLE 2:** Diagnostic yield in phenotypic subgroups and comparisons with an earlier review and other published systematic reviews.

	Diagnostic yield from current review, (95% CI)	Mellis et al. diagnostic yield, (95% CI)	Other systematic reviews, diagnostic yield, (95% CI)
Overall	27% (24%–30%)	31% (26%–36%)	
Multisystem	31% (26%–37%)	29% (22%–35%)	33% (27%–40%), Pauta et al. [14]
Skeletal	42% (34%–50%)	53% (42%–63%)	67%, isolated short long bones, Tse et al. [13] 84% (76%–90%), isolated dysplasia 33% (19%–49%), isolated dysostoses, Wang et al. [7] 48% (26%–70%), isolated short long bones 68% (58%–77%), short long bones with other skeletal abnormalities, Mone et al. [44]
CNS	23% (18%–27%) Isolated ACC: 16% (6%–26%)	17% (12%–22%)	22% (15%–31%), isolated single CNS abnormality 33% (22%–46%), ≥ 1 CNS only abnormality, Marchionni et al. [4] 32%, isolated ACC, Mustafa et al. [8]
Cardiac	11% (8%–14%)	11% (7%–16%)	11% (7%–15%), Mone et al. [33] 9% (7%–12%), Reilly et al. [9]
CAKUT	19% (13%–25%) Hyperechogenic kidneys 27% (10%–44%)	9% (5%–12%)	16% (6%–26%), Sonner et al. [10]
Respiratory	4% (0%–9%)	0	
Neuromuscular	46% (33%–59%)	37% (20%–54%)	
Hydrops	26% (19%–33%)	22% (14%–31%)	37% (30%–44%), isolated non‐immune hydrops, Al‐Kouatly et al. [12]
Gastrointestinal	5% (2%–8%)	2% (−4%–8%)	
Craniofacial	8% (5%–11%)	9% (1%–17%)	
IUGR	10% (5%–15%)	4% (−9%–17%)	12% (7%–18%), Pauta et al. [6]

Abbreviations: ACC, agenesis of corpus callosum; CAKUT, congenital abnormalities of the kidneys and urinary tract; CI, Confidence Interval; CNS, Central nervous system; IUGR, intrauterine growth restriction.

#### 3.9.1 Skeletal System

The overall diagnostic yield of sequencing in fetuses with isolated skeletal abnormalities was lower than previously reported (42% (95% CI 34%–50%) (Supporting Information S1: Figure S2)). Notably, the skeletal subgroup included two studies on isolated talipes [40, 45], and one study on isolated hemivertebrae [46]. When these studies were removed from the subgroup analysis, the overall diagnostic yield was 45% (95% CI 37%–53%).

#### 3.9.2 Central Nervous System

The diagnostic yield in fetuses with isolated central nervous system abnormalities is consistent with the current literature (23% (95% CI 18%–27%)). Ten studies in this subgroup focused on specific CNS abnormalities—six on abnormalities of the corpus callosum and one study each on posterior fossa malformations, cortical malformations, microcephaly and cerebellar vermis defects. The diagnostic yield in the CNS phenotypic group was comparable to that reported in a systematic review by Moradi et al. of 26.5% in brain abnormalities detected through MRI and ultrasound [12], although not specifically isolated CNS abnormalities in that review. Marchionni et al. reported a higher diagnostic yield of 33% in cases of one or more abnormalities confined to the CNS [5]. Importantly, lack of MRI confirmation can confound the classification of “isolated” as many CNS anomalies have subtle changes not detectable by ultrasound alone.

Of the six studies on corpus callosal abnormalities, Fetal MRI was used to confirm the diagnosis of isolated agenesis of the corpus callosum (ACC) for all fetuses in three studies [44, 47, 48], and for a proportion of cases in two studies [49, 50]. ACC was diagnosed with ultrasound only in one study without MRI confirmation [51]. The overall diagnostic yield for isolated corpus callosal abnormalities from these studies was 16% (95% CI 6%–26%) (Supporting Information S1: Figure S2). When a pooled analysis was performed only in studies with MRI confirmation of isolated ACC, the diagnostic yield was 27%. This is lower when compared to the yield of 32% reported for isolated ACC in the review by Mustafa et al. [9]. However, it was noted by the authors that not all studies reported the use of MRI to confirm that ACC was truly an isolated finding, which may have led to misclassification of some cases as isolated.

#### 3.9.3 Cardiovascular System

The pooled diagnostic yield of sequencing in fetuses with an isolated cardiac defect was 11% (95% CI 8%–14%), which is comparable with what is currently reported in the literature [10, 52]. Included in the cardiac subgroup of this review were five studies which focused on specific isolated cardiac phenotypes. The diagnostic yields were as follows: 0% [53], 11% [54] and 18% [55] from three studies on conotruncal defects, 23% from one study on isolated dextrocardia [56], and 8% from one study on isolated ventricular septal defect [57].

#### 3.9.4 Congenital Abnormalities of Kidneys and Urinary Tract (CAKUT)

Included in the CAKUT subgroup were three studies representing 25 fetuses focused specifically on isolated hyperechogenic kidneys. The pooled diagnostic yield in hyperechogenic kidneys was 27% (95% CI 10%–44%), which is lower than the 51% previously reported in isolated echogenic kidneys [11].

#### 3.9.5 Fetal Growth Restriction

Although this review focused on the incremental yield of sequencing in fetuses with a structural abnormality, several cohorts included a subgroup of fetuses with isolated fetal growth restriction (FGR). Three additional studies were identified from a separate literature search on prenatal sequencing in isolated growth restriction over the same time period [58, 59, 60]. Combined with four papers from the original review [61, 62, 63, 64], the pooled diagnostic yield of sequencing in isolated FGR was 10% (95% CI 5%–15%) (Supporting Information S1: Figure S2), consistent with the 12% yield reported in a recent systematic review of isolated FGR [7]. Notably, information on Doppler studies was not provided in many records included in our review, making it difficult to distinguish between FGR with placental and non‐placental causes. Mone et al. reported a lower diagnostic yield of 4% in isolated FGR with evidence of placental insufficiency [65], highlighting the importance of distinguishing between these groups.

#### 3.9.6 Increased Nuchal Translucency (NT)

Ten studies in this review focused on fetuses with an increased NT, but we did not perform subgroup analysis as this is not technically a structural malformation. Furthermore, inclusion criteria varied significantly, ranging from NT > 95th centile to NT > 6.5 mm. Lastly, several studies did not specify whether additional abnormalities were seen on subsequent scans or whether it was a transient finding, which is of particular importance given the knowledge that the diagnostic yield of sequencing is highly dependent on whether the increased NT remains an isolated finding throughout pregnancy [4, 6, 17].

#### 3.9.7 Incremental Yield of GS

Of the 16 studies using GS, this was used for a proportion of the cohort in six studies, and for the entire cohort in 10 studies. Data from these 10 studies represented 935 fetuses with structural abnormalities. From these studies, clinically relevant diagnoses were made in nine cases (Table 3) which would have been missed by the sequential testing strategy of CMA followed by ES, demonstrating an increase in diagnostic yield above ES of 1% (9/935), consistent with current literature [66]. Five studies reported variants of uncertain significance, which ranged from 4% −42%. Two studies with high VUS rates of 42% and 20% were conducted as a proband‐only analysis [67, 68].

**TABLE 3:** Additional diagnoses from studies which performed concurrent CNV and SNV analysis.

Author, year	Sequencing method	Diagnosis
Zhou (2021)	GS trio compared with sequential ES after non‐diagnostic CMA	Dual diagnosis of 11.7 Mb duplication of 8p22‐8p23.3 and de novo pathogenic missense variant in *GJA8* related to phenotype
Yang (2022)	GS solo after non‐diagnostic low‐pass GS	3 cases of small pathogenic intragenic CNVs in *KAT6A*, *NF1* and *TM4SF20* (size range 1.3–58.8 kb) in CNS abnormalities
Lowther (2023)	GS trio compared with karyotype/CMA ± ES	1.3 kb pathogenic intragenic deletion of *MED13L* with lymphatic malformation Compound heterozygous variant pair of missense variant in trans with 143 kb intragenic exonic duplication in *DYNC2H1*in fetus with short‐rib thoracic dysplasia
Hu (2023)	GS solo or trio compared with CMA	2.1 kb pathogenic deletion in *COL4A2* in fetus with porencephaly
Qi (2024)	GS trio after non‐diagnostic CMA and ES	Compound heterozygous variant pair of pathogenic nonsense variant and likely pathogenic 5.16 kb in *BBS9* in fetus with bilateral echogenic kidneys and polydactyly Likely pathogenic 3.03 kb deletion in *FGF8* in fetus with holoprosencephaly

Abbreviations: CMA, chromosomal microarray analysis; CNV, copy number variant; ES, exome sequencing; GS, genome sequencing; SNV, single nucleotide variant.

## 4 Discussion

The results from this systematic review further reinforce the role of prenatal sequencing in the diagnosis of fetal structural abnormality, providing an additional diagnostic yield of 27% following a non‐diagnostic CMA across a range of indications. Importantly, the comparisons between the studies from this and the earlier review demonstrate several trends: (1) an increase in the number of prenatal sequencing studies, (2) an increase in genome sequencing, (3) more studies on specific phenotypic subgroups/phenotypes, and (4) more exploratory studies on less evidenced phenotypes (Table 1).

Acknowledging the limited clinical value of a pooled diagnostic yield across a wide range of inclusion criteria, this review performed several subgroup analyses aiming to provide a more specific yield per indication. Previously published literature has emphasized the importance of pre‐selecting cases with a higher likelihood of a monogenic disorder and its impact on diagnostic yield [1, 2]. Results from this review further reinforce this by demonstrating a statistically significant difference in diagnostic yield between selected and unselected cohorts (40% vs. 17%, *p* < 0.0001). In countries where genetic testing is publicly funded, national guidance on inclusion criteria for prenatal sequencing is essential to ensure cost‐effectiveness. With the current literature, sufficient evidence exists to support the role of sequencing in specific indications with known high diagnostic yields, and these are typically included in national criteria [18]. However, as more studies are performed for exploratory indications, regular review of this data is needed to determine the need for the revision of inclusion criteria to further improve diagnostic yield. Additionally, results from these studies can help guide decisions about testing for indications which fall outside the boundaries of well‐evidenced criteria. However, data from small studies must of course be interpreted with caution.

While there is a clear benefit in sequencing fetuses with multi‐system anomalies, the evidence is less clear in isolated single system anomalies owing to smaller sample sizes. With an increasing number of studies on prenatal sequencing, stronger conclusions may be drawn from pooled sample sizes, but the data may also become more heterogeneous. This is demonstrated by the diagnostic yield from the isolated skeletal subgroup in this study (42%), which appears lower than previously reported (53%) [1] (Table 2). This apparent decrease in diagnostic yield is likely to be explained by the inclusion of studies under the ‘skeletal’ subgroup performed in fetuses with anomalies such as talipes or hemivertebrae, which are known to have a lower likelihood of a genetic etiology than fetuses with short long bones and suspected skeletal dysplasias. This emphasizes the fact that within phenotypic subgroups, current data already suggest that certain isolated abnormalities have a higher likelihood of being due to a single gene disorder. Hence, while studies performed in a phenotypic group may provide stronger evidence due to larger sample sizes, the heterogeneity of the included population may mask the true value of sequencing in specific anomalies. Furthermore, few studies describe the standard of scanning delivered, or the extent of other imaging modalities used. For example, MRI in CNS anomalies is usually required to determine if other CNS abnormalities (e.g., cortical changes) are present or if an anomaly is truly isolated, but the use of MRI is not usually consistently reported. In the review of sequencing of fetuses with isolated short long bones [65], careful review of the papers reporting “isolated” short long bones found that this included many cases where standard fetal anomaly scanning should have detected additional features suggesting a genetic diagnosis.

Our review has demonstrated a shift of focus in the literature from overall diagnostic yields to the role of prenatal sequencing in specific phenotypic groups and exploratory indications. While these studies are needed, it is important to determine whether the findings from these studies can be appropriately applied to individual clinical practice. When interpreting the diagnostic yield of a study, it is first important to consider the method of case‐selection and inclusion criteria. A higher diagnostic yield is expected when there is case selection based on evidenced criteria or when there is an increased pre‐test probability of a genetic disorder due to factors such as consanguinity and a previously affected pregnancy [1, 2, 13]. Next, although more costly, the trio approach to sequencing is recommended in the prenatal setting [69] to reduce the number of VUS reported and the time required for variant curation. This is reflected by the apparently lower VUS rates in the trio studies reported in this review. Additionally, as discussed above, performing CNV analysis using sequencing data may detect variants missed by CMA. With improved bioinformatic pipelines, this is made increasingly possible, especially with GS. Although a clear additional benefit of GS over ES remains to be demonstrated in the prenatal setting, attractive aspects of GS such as faster library preparation, decreasing costs, and shorter turnaround times may lead to a continued increase in its use. Lastly, variant reporting should follow internationally recommended guidelines to ensure standardization of what is considered ‘diagnostic’ before the true diagnostic yield can be ascertained from a study for clinical use.

### 4.1 Strengths and Limitations

This review presents updated and detailed diagnostic yields of prenatal sequencing across various indications. Importantly, the comparison of results from this review and its earlier version also demonstrates a shift in focus from targeted sequencing to broader approaches applied to phenotypic subgroups. The main limitation of this study is the heterogeneity in case selection, sequencing approaches, analytic methods, variant interpretation, and reporting, which may limit the clinical application of pooled diagnostic yields. Heterogeneity of data across studies is expected as sequencing becomes more accessible worldwide. To overcome the limitations that this heterogeneity poses, knowledge of the factors affecting diagnostic yield is necessary to critically assess individual study data before application to clinical practice. This study, together with our bioinformatics review [70], aims to provide a framework to do this. Lastly, the studies included in this review may not be a true reflection of global prenatal sequencing practices as the reports from countries included here only represented slightly more than half of the countries identified as offering prenatal sequencing in a recent global survey [18].

In summary, the findings from our study further support the role of sequencing in prenatal diagnosis. Results from further well‐designed sequencing studies on fetuses with specific phenotypes are needed to enhance the robustness of current knowledge to guide clinical practice. This review comprehensively demonstrates the breadth of existing data, as well as highlights the remaining gaps in knowledge.

## Funding

K.M.X.L. is on a clinical fellowship at Great Ormond Street Hospital for Children Foundation Trust funded by the Human Manpower Development Program, awarded by the Ministry of Health Singapore. E.S. and L.S.C. are funded by the NIHR Biomedical Research Centre at Great Ormond Street Hospital. The views expressed are those of the author and not necessarily those of the NHS, the NIHR, or the Department of Health and Social Care.

## Ethics Statement

The authors have nothing to report.

## Conflicts of Interest

The authors declare no conflicts of interest.

## Supporting information


Supporting Information S1

**Table S1:** Summary of 89 studies included in the updated systematic review.

## Data Availability

The data that supports the findings of this report are available from the authors upon a reasonable request.
