# Supplementary material for: Diagnostic Yield of Sequencing for Prenatal Diagnosis of Fetal Structural Anomalies: An Updated Systematic Review
Source: Prenat Diagn. 2026 Mar 2;46(5-6):756–70. doi: 10.1002/pd.70112 (PMC13170049; doi:10.1002/pd.70112)
Supplement: Supplementary file 1 — Supporting Information S1 [file PD-46-756-s001.pptx]

## Slide 1
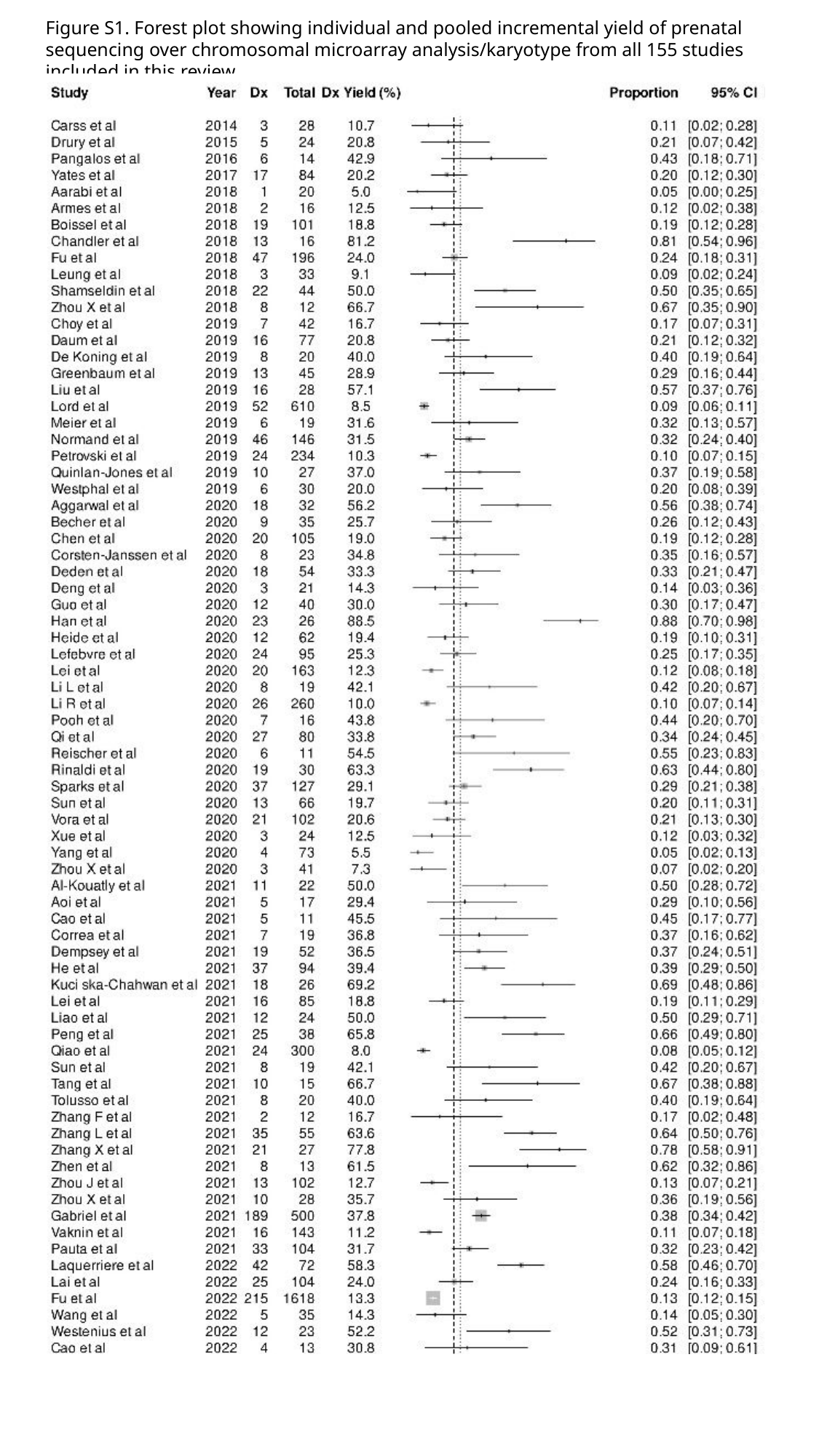

Figure S1. Forest plot showing individual and pooled incremental yield of prenatal sequencing over chromosomal microarray analysis/karyotype from all 155 studies included in this review

## Slide 2
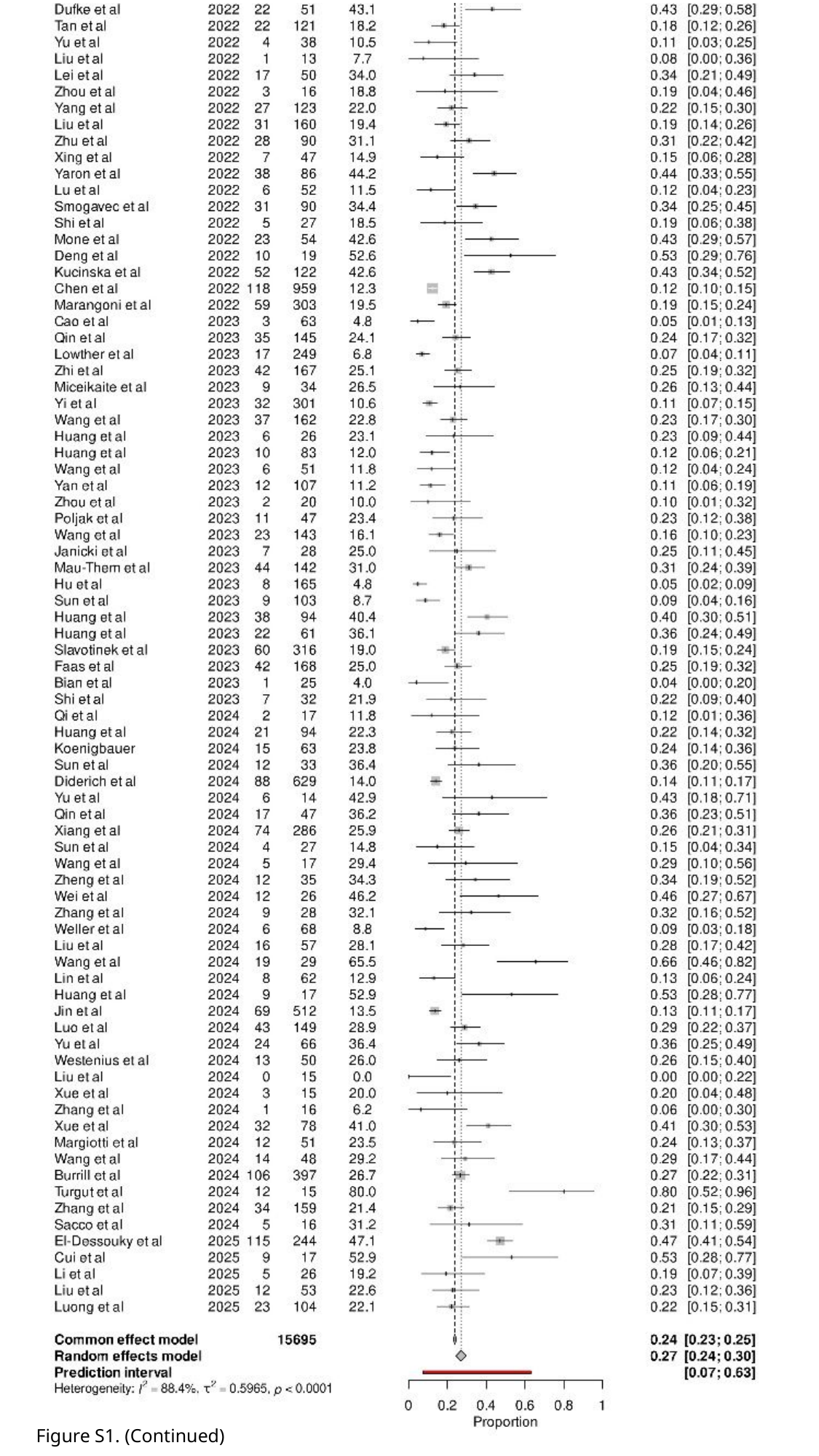

Figure S1. (Continued)

## Slide 3
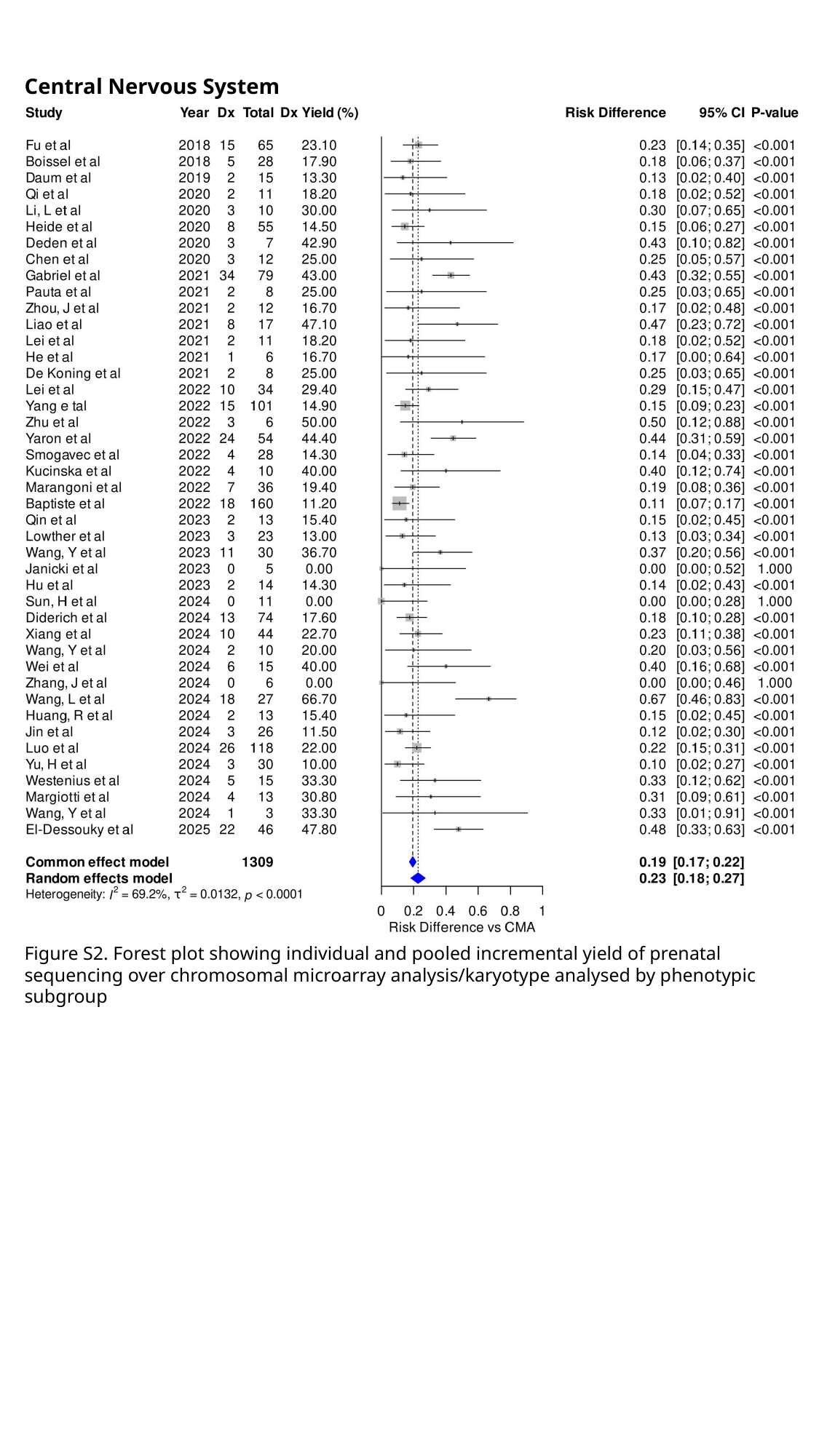

Central Nervous System
Figure S2. Forest plot showing individual and pooled incremental yield of prenatal sequencing over chromosomal microarray analysis/karyotype analysed by phenotypic subgroup

## Slide 4
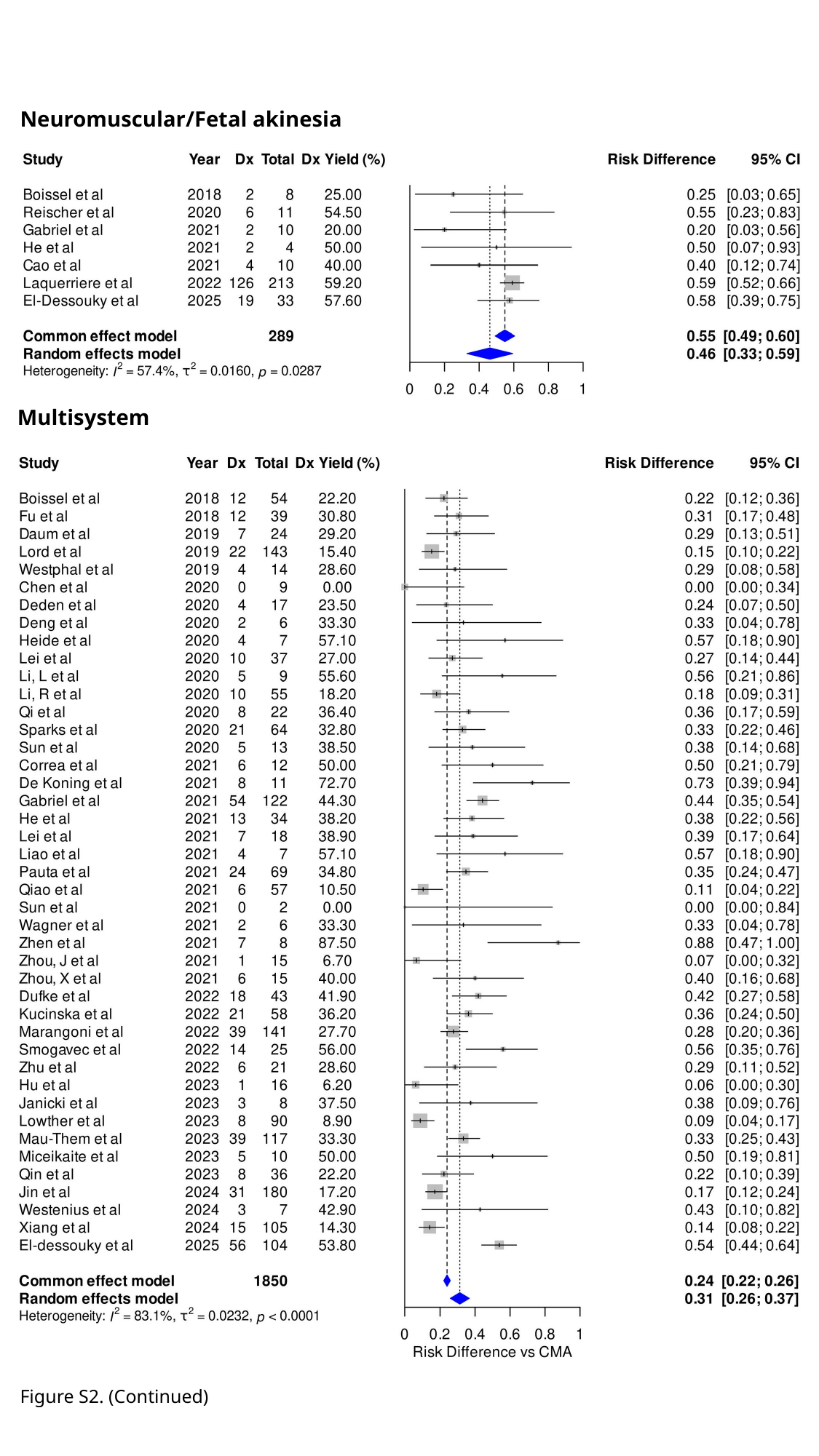

Neuromuscular/Fetal akinesia
Multisystem
Figure S2. (Continued)

## Slide 5
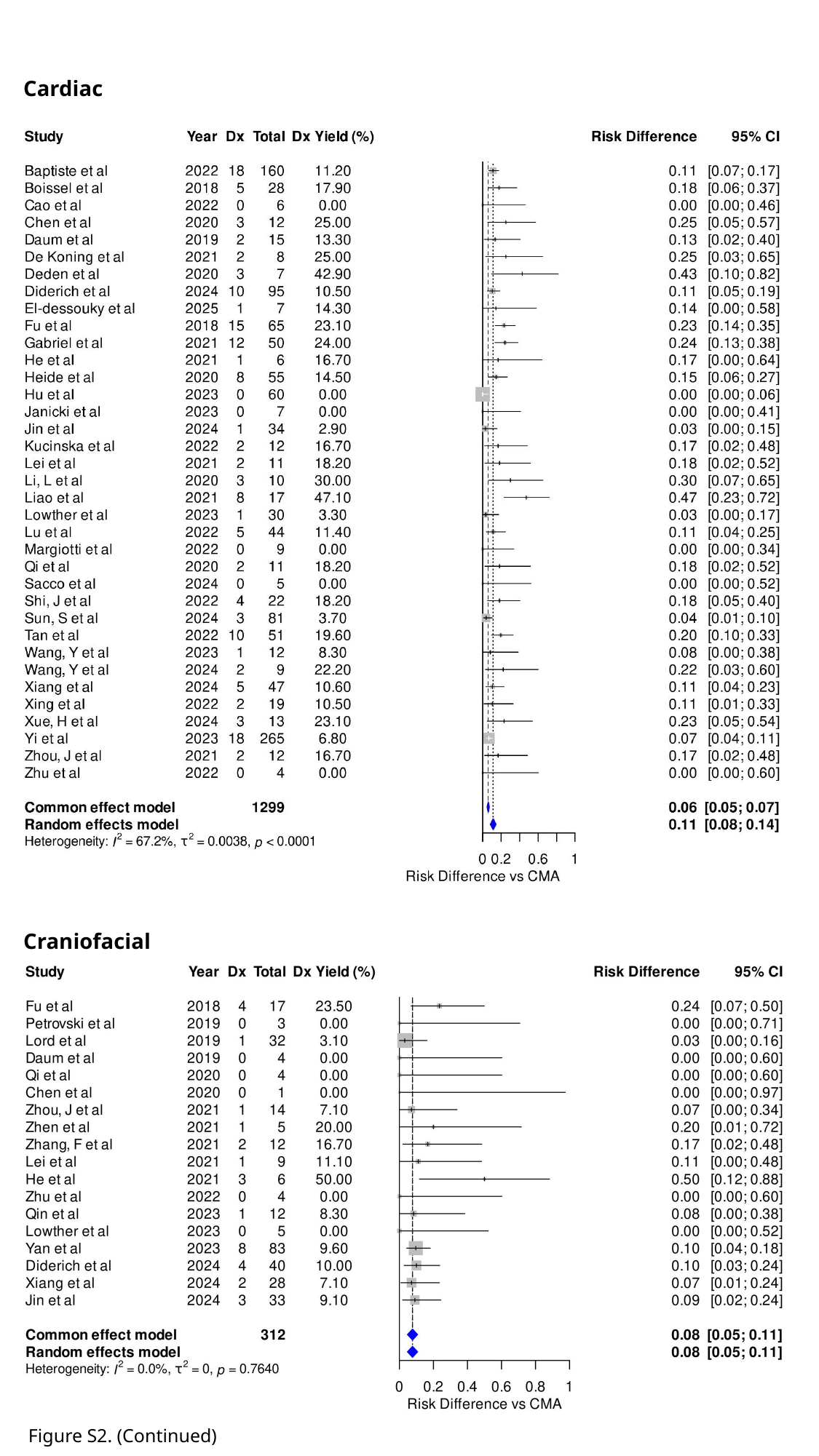

Cardiac
Craniofacial
Figure S2. (Continued)

## Slide 6
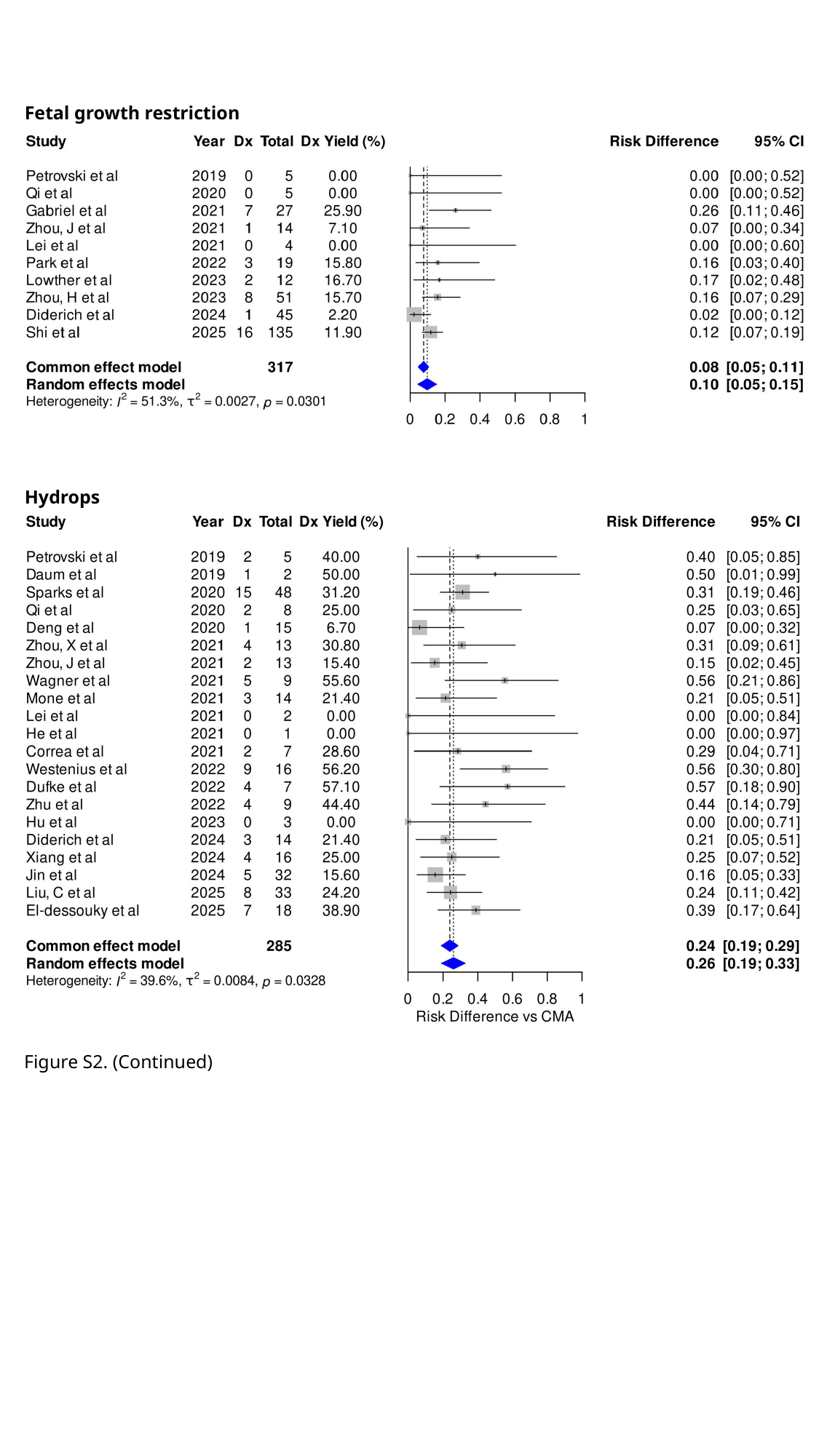

Fetal growth restriction
Hydrops
Figure S2. (Continued)

## Slide 7
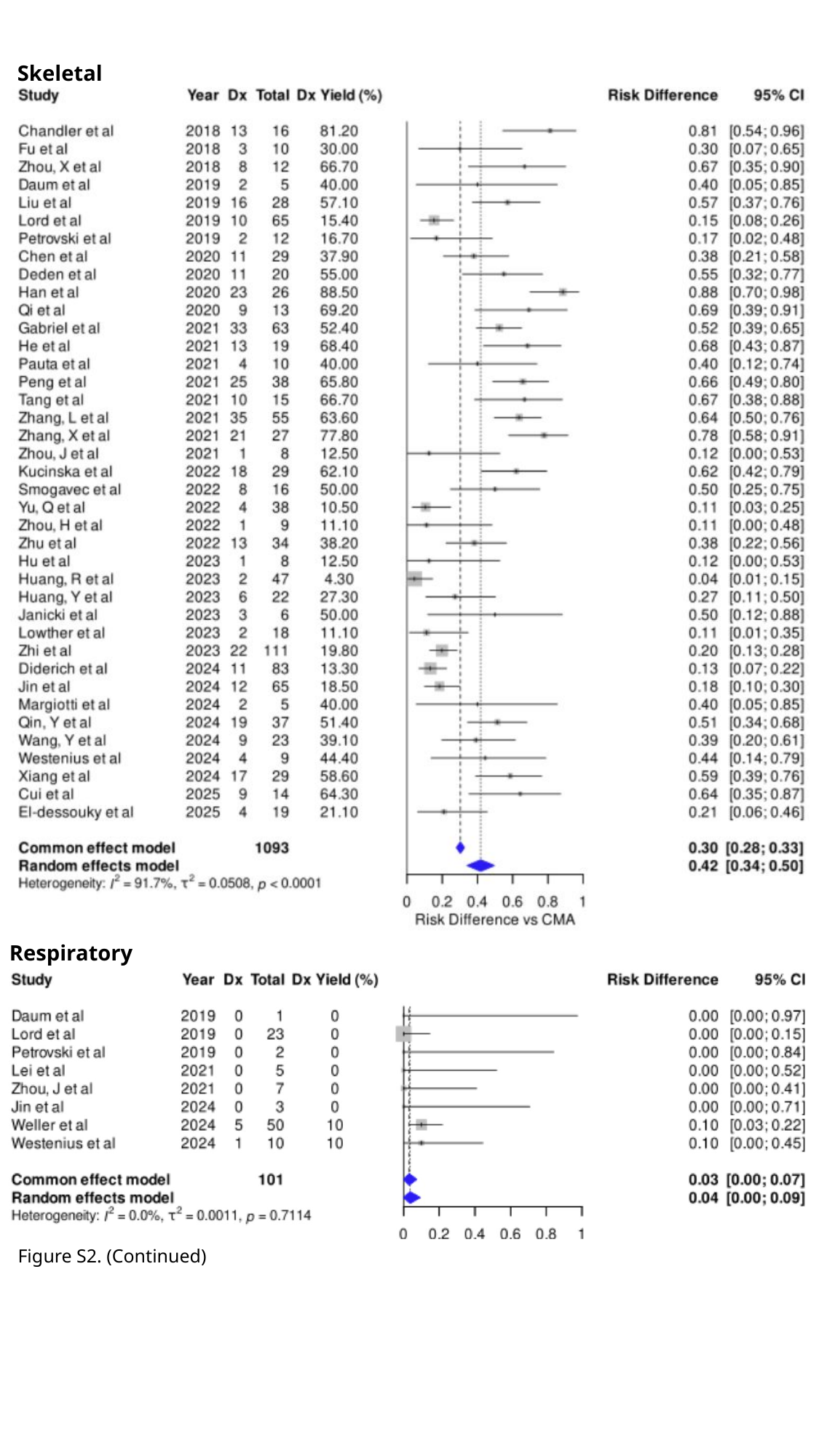

Skeletal
Respiratory
Figure S2. (Continued)

## Slide 8
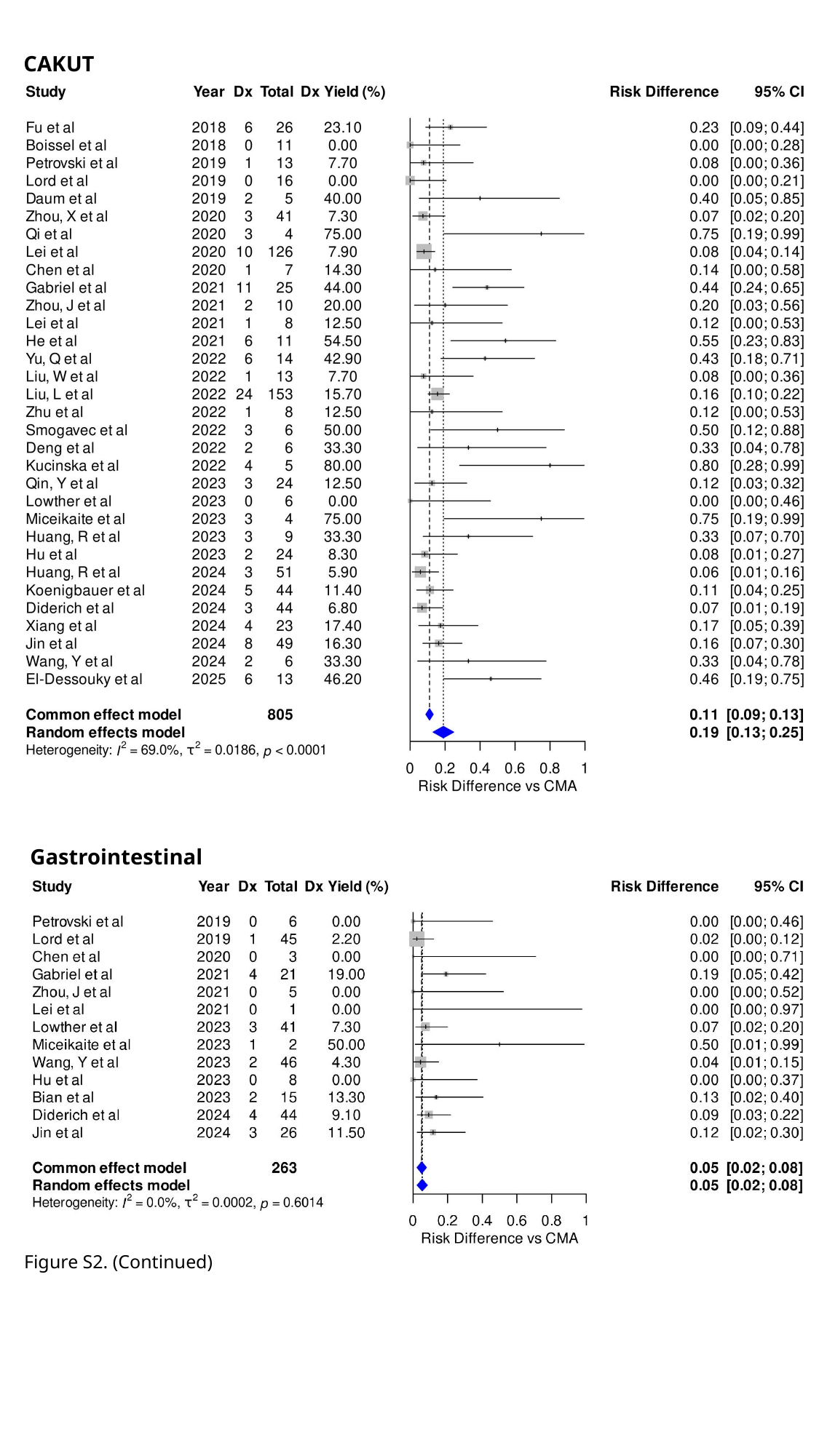

CAKUT
Gastrointestinal
Figure S2. (Continued)

## Slide 9
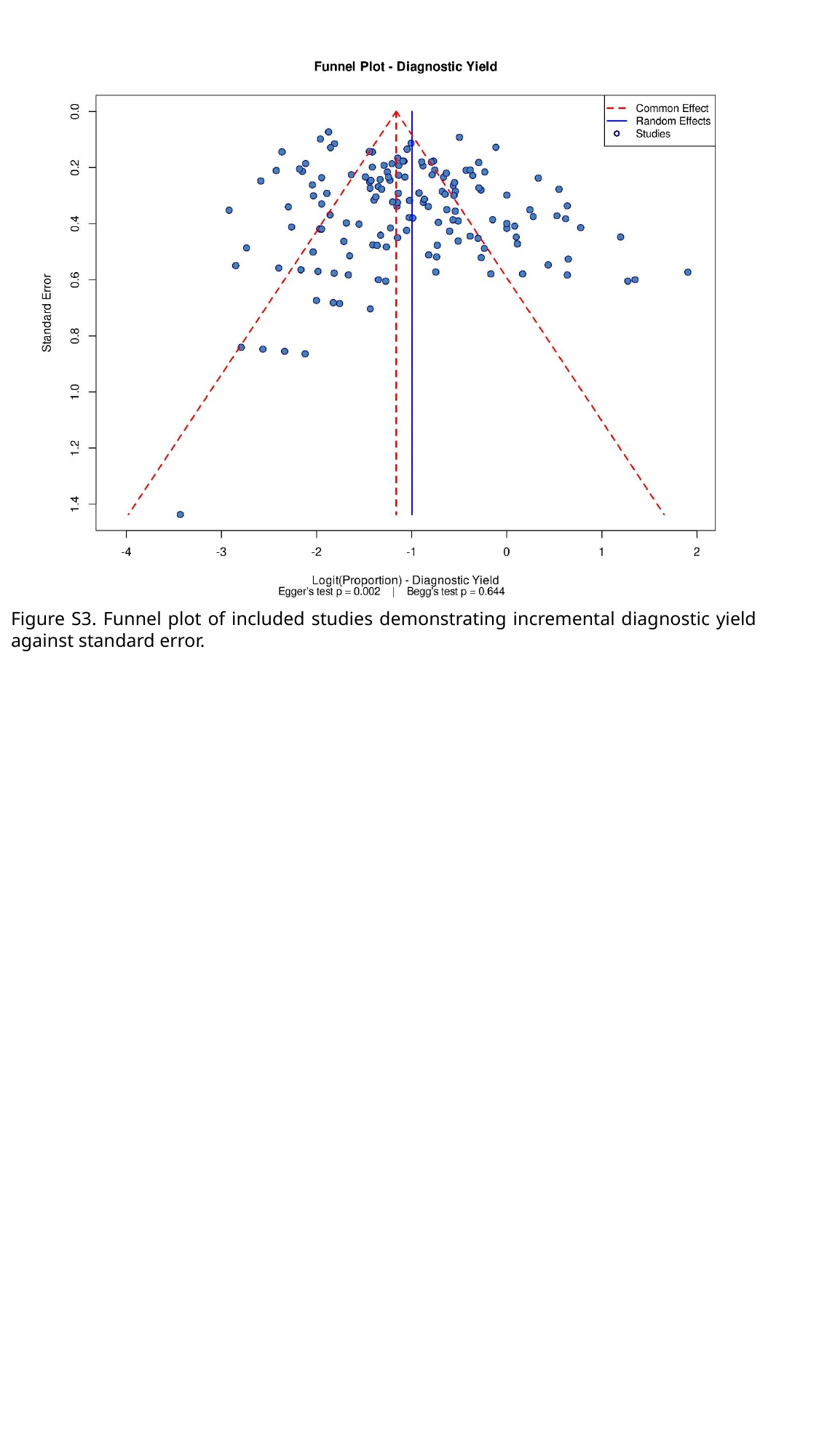

Figure S3. Funnel plot of included studies demonstrating incremental diagnostic yield against standard error.
