# Supplementary material for: Diagnostic Yield of Sequencing for Prenatal Diagnosis of Fetal Structural Anomalies: An Updated Systematic Review
Source: Prenat Diagn. 2026 Mar 2;46(5-6):756–70. doi: 10.1002/pd.70112 (PMC13170049; doi:10.1002/pd.70112)
Supplement: Supplementary file 2 — Table S1: Summary of 89 studies included in the updated systematic review. [file PD-46-756-s002.pdf]

Table S1. Summary of 89 studies included in the updated systematic review

	Study Title	Author, year	Country	Probands sequenced	Inclusion criteria	Selection classification	Sequencing approach and analysis	Diagnostic variants (%)	VUS (%)	Incidental findings (%)
1	Whole-exome sequencing applications in prenatal diagnosis of fetal bowel dilatation.	Bian et al, 2023	China	25	Gastro-intestinal	Exploratory	ES Trio	1/25 (4.0%)	1/25 (4.0%)	ND
2	Whole Exome Sequencing in a Population of Fetuses With Structural Anomalies	Burrill et al, 2024	USA	397	Major Fetal Abnormality not specified to one system	Selected	ES Trio	106/397 (26.7%)	67/397 (16.9%)	ND
3	Exploring the diagnostic utility of genome sequencing for fetal congenital heart defects	Cao et al, 2022	Hong Kong	13	Cardiac	Exploratory	GS Trio	4/13 (30.8%)	3/13 (23.1%)	ND<br><br>4 SF reported
4	Prenatal whole-exome sequencing in fetuses with increased nuchal translucency	Cao et al, 2023	China	63	Increased Nuchal Translucency – isolated	Exploratory	ES Trio	3/63 (4.8%)	5/63 (7.9%)	ND
5	Clinical efficiency of simultaneous CNV-seq and whole-exome sequencing for testing fetal structural anomalies	Chen et al, 2022	China	959	Major Fetal Abnormality not specified to one system	Unselected	ES Trio	118/959 (12.3%)	ND	ND
6	Analysis of a Series of 26 Cases With Prenatal Skeletal Dysplasia via Multiplatform Genetic Detection	Cui et al, 2025	China	17	Skeletal	Selected	ES Solo	9/17 (52.9%)	6/17 (35.3%)	ND
7	Prenatal diagnosis and outcome of fetal hyperechogenic kidneys in the era of antenatal next-generation sequencing	Deng et al, 2022	China	19	CAKUT – hyper-echogenic kidneys	Selected	ES Trio	10/19 (52.6%)	3/19 (15.8%)	ND
8	The High Diagnostic Yield of Prenatal Exome Sequencing Followed by 3400 Gene Panel Analysis in 629 Ongoing	Diderich et al, 2024	Netherlands	629	Major Fetal Abnormality not specified to one system	Selected	ES Trio with fetal gene panel (3400 genes)	88/629 (14.0%)	ND	ND

	Pregnancies With Ultrasound Anomalies									
9	A single center experience of prenatal parent-fetus trio exome sequencing for pregnancies with congenital anomalies	Dufke et al, 2022	Germany	51	Major Fetal Abnormality not specified to one system	Selected	ES Trio	22/51 (43.1%)	1/51 (2.0%)	ND<br><br>1 SF reported
10	Integrating Prenatal Exome Sequencing and Ultrasonographic Fetal Phenotyping for Assessment of Congenital Malformations: High Molecular Diagnostic Yield and Novel Phenotypic Expansions in a Consanguineous Cohort	El-Dessouky et al, 2025	Egypt	244	Major Fetal Abnormality not specified to one system	Selected	ES Solo	115/244 (47.1%)	84/244 (34.4%)	15/244 (6.1%)
11	All-in-one whole exome sequencing strategy with simultaneous copy number variant, single nucleotide variant and absence-of-heterozygosity analysis in fetuses with structural ultrasound anomalies: A 1-year experience	Faas et al, 2023	Nether-lands	168	Major Fetal Abnormality not specified to one system	Unselected	ES Trio with phenotype-specific panel	42/168 (25.0%)	ND	8/168 (4.8%)
12	Application of exome sequencing for prenatal diagnosis of fetal structural anomalies: clinical experience and lessons learned from a cohort of 1618 fetuses	Fu et al, 2022	China	1618	Major Fetal Abnormality not specified to one system	Unselected	ES Trio	215/1618 (13.3%)	123/1618 (7.6%)	8/1618 (0.5%)<br><br>13 SF reported
13	Trio exome sequencing is highly relevant in prenatal diagnostics	Gabriel et al, 2021	Germany	500	Major Fetal Abnormality not specified to one system	Unselected	ES Trio	189/500 (37.8%)	14/500 (2.8%)	9/500 (1.8%)
14	Whole genome sequencing vs chromosomal microarray	Hu et al, 2023	China	165	Major Fetal Abnormality not specified to one system	Unselected	GS Solo or Trio	8/165 (4.8%)	ND	3/165 (1.8%)

	analysis in prenatal diagnosis									
15	Prenatal diagnosis in the fetal hyperechogenic kidneys: assessment using chromosomal microarray analysis and exome sequencing	Huang, R et al, 2023	China	26	CAKUT – hyperechogenic kidneys	Selected	ES Trio	6/26 (23.1%)	6/26 (23.1%)	ND
16	Whole exome sequencing improves genetic diagnosis of fetal clubfoot	Huang, R et al, 2023	China	83	Skeletal – talipes	Exploratory	ES Trio	10/83 (12.0%)	8/83 (9.6%)	ND
17	Prenatal diagnosis of polycystic renal diseases: diagnostic yield, novel disease-causing variants, and genotype-phenotype correlations	Huang, R et al, 2024	China	94	CAKUT	Exploratory	ES Solo	21/94 (22.3%)	8/94 (8.5%)	ND
18	Retrospective analysis of the prognostic factors of fetal corpus callosum dysplasia	Huang, R et al, 2024	China	17	Central Nervous System - ACC	Selected	ES Trio	9/17 (52.9%)	ND	ND
19	Whole-exome sequencing in deceased fetuses with ultrasound anomalies: a retrospective analysis	Huang, W et al, 2023	China	61	Major Fetal Abnormality not specified to one system	Selected	ES Solo or Trio	22/61 (36.1%)	6/61 (9.8%)	ND
20	Exome sequencing in fetuses with short long bones detected by ultrasonography: A retrospective cohort study	Huang, Y et al, 2023	China	94	Skeletal – short long bones	Selected	CES Trio	38/94 (40.4%)	5/94 (5.3%)	ND
21	Implementation of Exome Sequencing in Prenatal Diagnostics: Chances and Challenges	Janicki et al, 2023	Belgium	28	Major Fetal Abnormality not specified to one system	Selected	ES Trio with phenotype-specific panel	7/28 (25.0%)	ND	1/28 (3.6%)
22	Molecular diagnostic yield of exome sequencing in a Chinese cohort of 512 fetuses with anomalies	Jin et al, 2024	China	512	Major Fetal Abnormality not specified to one system	Unselected	ES Trio	69/512 (13.5%)	ND	ND
23	Spectrum of congenital anomalies of the kidney and urinary tract (CAKUT) including renal	Koenigbauer et al, 2024	Germany	63	CAKUT	Exploratory	ES Trio	15/63 (23.8%)	2/63 (3.2%)	5/63

	parenchymal malformations during fetal life and the implementation of prenatal exome sequencing (WES)									
24	Implementation of Exome Sequencing in Prenatal Diagnosis and Impact on Genetic Counseling: The Polish Experience	Kucinska et al, 2022	Poland	122	Major Fetal Abnormality not specified to one system	Unselected	ES Solo	52/122 (42.6%)	39/122 (32.0%)	ND
25	Application of Prenatal Whole Exome Sequencing for Structural Congenital Anomalies—Experience from a Local Prenatal Diagnostic Laboratory	Lai et al, 2022	Hong Kong	104	Major Fetal Abnormality not specified to one system	Selected	ES Trio	25/104 (24.0%)	8/104 (7.7%)	ND
26	Phenotypic spectrum and genomics of undiagnosed arthrogryposis multiplex congenita	Laquerriere et al, 2022	France	72	Neuro-muscular – Fetal Akinesia	Selected	ES Solo	42/72 (58.3%)	ND	ND
27	Prenatal exome sequencing in fetuses with callosal anomalies	Lei, T et al, 2022	China	50	Central Nervous System – ACC	Selected	ES Trio	17/50 (34.0%)	ND	ND
28	Prenatal diagnosis and clinical pregnancy outcome of fetuses with conotruncal defects in a Chinese cohort	Li, M et al, 2025	China	26	Cardiac	Exploratory	ES Solo	5/26 (19.2%)	4/26 (15.4%)	ND
29	Contribution of genetic variants to congenital heart defects in both singleton and twin fetuses: a Chinese cohort study	Lin et al, 2024	China	62	Cardiac	Exploratory	ES Solo or Trio	8/62 (12.9%)	ND	ND
30	Exome sequencing for nonimmune hydrops fetalis and clinical utility of data reanalysis	Liu, C et al, 2025	China	53	Hydrops	Selected	CES Trio	12/53 (22.6%)	7/53 (13.2%)	ND
31	Prenatal evaluation of genetic variants in fetuses with small head	Liu, J et al, 2024	China	57	Central Nervous System – microcephaly	Exploratory	ES trio with DDG2P panel (then ‘open	16/57 (28.1%)	11/57 (19.3%)	ND

	circumference: A single-center retrospective study						exome' if panel non-diagnostic			
32	Genetic diagnosis of common fetal renal abnormalities detected on prenatal ultrasound	Liu, L et al, 2022	China	160	CAKUT	Exploratory	ES Trio	31/160 (19.4%)	ND	ND
33	The Evaluation of Genetic Diagnosis on High-Risk Fetal CAKUT	Liu, W et al, 2022	China	13	CAKUT	Exploratory	ES Solo	1/13 (7.7%)	3/13 (23.1%)	ND
34	Prenatal Chromosomal Microarray Analysis and Whole-Exome Sequencing in Fetuses with Thickened Nuchal Translucency	Liu, X et al, 2024	China	15	Increased Nuchal Translucency	Exploratory	ES Trio	0/15	6/15 (40.0%)	ND
35	Systematic evaluation of genome sequencing for the diagnostic assessment of autism spectrum disorder and fetal structural anomalies	Lowther et al, 2023	USA	249	Major Fetal Abnormality not specified to one system	Unselected	GS Trio with fetal anomaly panel (2535 genes)	17/249 (6.8%)	ND	ND
36	Estimating the frequency of causal genetic variants in foetuses with congenital heart defects: a Chinese cohort study	Lu et al, 2022	China	52	Cardiac	Exploratory	ES Trio	6/52 (11.5%)	12/52 (23.1%)	ND
37	Application of Whole-Exome Sequencing in the Prenatal Diagnosis of Foetuses With Central Nervous System Abnormalities	Luo et al, 2024	China	149	Central Nervous System	Selected	ES Trio	43/149 (28.9%)	ND	ND
38	Combined Cell-Free DNA Screening for Aneuploidies and Selected Single-Gene Disorders for Pregnancies With Sonographically Detected Fetal Anomalies: Detection Rate and Residual Risk	Luong et al, 2025	Vietnam	104	Major Fetal Abnormality not specified to one system	Unselected	ES Solo	23/104 (22.1%)	ND	ND
39	Implementation of fetal clinical exome	Marangoni et al, 2022	Belgium	303	Major Fetal Abnormality not	Unselected	ES Trio	59/303 (19.5%)	20/303 (6.6%)	7/303 (2.3%)

	sequencing: Comparing prospective and retrospective cohorts				specified to one system					
40	Prenatal Diagnosis by Trio Clinical Exome Sequencing: Single Center Experience	Margiotti et al, 2024	Italy	51	Major Fetal Abnormality not specified to one system	Unselected	CES Trio	12/51 (23.5%)	ND	ND
41	Prenatal diagnosis by trio exome sequencing in fetuses with ultrasound anomalies: A powerful diagnostic tool	Mau-Them et al, 2023	France	142	Major Fetal Abnormality not specified to one system	Selected	ES Trio	44/142 (31.0%)	13/142 (9.2%)	2/142 (1.4%)
42	Comprehensive prenatal diagnostics: Exome versus genome sequencing	Miceikaite et al, 2023	Denmark	34	Increased Nuchal Translucency	Exploratory	ES or GS Trio	9/34 (26.5%)	1/34 (2.9%)	ND<br><br>3 SF reported
43	Evolving fetal phenotypes and clinical impact of progressive prenatal exome sequencing pathways: cohort study	Mone et al, 2022	UK	54	Major Fetal Abnormality not specified to one system	Selected	CES Trio	23/54 (42.6%)	1/54 (1.9%)	2/54 (3.7%)
44	Next-Generation Sequencing Gene Panels and "Solo" Clinical Exome Sequencing Applied in Structurally Abnormal Fetuses	Pauta et al, 2021	Spain	104	Major Fetal Abnormality not specified to one system	Unselected	CES Solo	33/104 (31.7%)	5/104 (4.8%)	ND
45	Prenatal exome sequencing and impact on perinatal outcome: cohort study	Poljak et al, 2023	UK	47	Major Fetal Abnormality not specified to one system	Selected	ES Trio	11/47 (23.4%)	2/47 (4.3%)	1/47 (2.1%)
46	Whole-genome sequencing analysis in fetal structural anomalies: novel phenotype–genotype discoveries	Qi et al, 2024	China	17	Major Fetal Abnormality not specified to one system	Selected	GS Trio	2/17 (11.8%)	ND	ND
47	Application of Whole Exome Sequencing in the Genetic Diagnosis of Prenatal Ultrasound Abnormalities	Qin, L et al, 2024	China	47	Major Fetal Abnormality not specified to one system	Unselected	ES Trio	17/47 (36.2%)	ND	ND

48	Prenatal whole-exome sequencing for fetal structural anomalies: a retrospective analysis of 145 Chinese cases	Qin, Y et al, 2023	China	145	Major Fetal Abnormality not specified to one system	Unselected	ES Solo or Trio	35/145 (24.1%)	13/145 (9%)	4/145 (2.8%)
49	The evolving genetic etiology of conotruncal anomalies	Sacco et al, 2024	UK	16	Cardiac – conotruncal	Exploratory	ES or GS Trio	5/16 (31.3%)	ND	1/16 (6.3%)
50	Diagnostic yield of whole exome data in fetuses aborted for conotruncal malformations	Shi, J et al, 2022	China	27	Cardiac – conotruncal	Exploratory	ES Solo with custom CHD panel (252 genes)	5/27 (18.5%)	Unclear	ND
51	Clinical utility of chromosomal microarray analysis and whole exome sequencing in foetuses with oligohydramnios	Shi, X et al, 2023	China	32	CAKUT – oligo-hydramnios	Exploratory	ES Trio	7/32 (21.9%)	1/32 (3.1%)	ND
52	Diagnostic yield of pediatric and prenatal exome sequencing in a diverse population	Slavotinek et al, 2023	USA	316	Major Fetal Abnormality not specified to one system	Unselected	ES Trio	60/316 (19.0%)	20/316 (6.3%)	0
53	Singleton exome sequencing of 90 fetuses with ultrasound anomalies revealing novel disease-causing variants and genotype–phenotype correlations	Smogavec et al, 2022	Austria	90	Major Fetal Abnormality not specified to one system	Unselected	ES or CES Solo	31/90 (34.4%)	3/90 (3.3%)	2/90 (2.2%)<br><br>1 SF reported
54	Fetal agenesis of the corpus callosum: Clinical and genetic analysis in a series of 40 patients	Sun, H et al, 2024	China	33	Central Nervous System – ACC	Selected	ES Trio	12/33 (36.4%)	ND	ND
55	Genomic insights into prenatal diagnosis of congenital heart defects: value of CNV-seq and WES in clinical practice	Sun, S et al, 2024	China	27	Cardiac	Exploratory	ES Solo or Trio	4/27 (14.8%)	3/27 (11.1%)	6/27 (22.2%)
56	Genetic examination for fetuses with increased nuchal translucency by exome sequencing	Sun, Y et al, 2023	China	103	Increased Nuchal Translucency	Exploratory	ES Trio	9/103 (8.7%)	3/103 (2.9%)	3/103 (2.9%)

57	Genetic Diagnostic Yield and Novel Causal Genes of Congenital Heart Disease	Tan et al, 2022	China	121	Cardiac	Exploratory	ES Solo	22/121 (18.2%)	ND	ND
58	Clinical and molecular characteristics of 26 fetuses with lethal multiple congenital contractures	Turgut et al, 2024	Turkey	15	Neuro-muscular – multiple contractures	Selected	ES Solo	12/15 (80.0%)	3/15 (20.0%)	ND
59	High rate of abnormal findings in Prenatal Exome Trio in low risk pregnancies and apparently normal fetuses	Vaknin et al, 2021	Israel	143	Major Fetal Abnormality not specified to one system	Selected	CES Trio	16/143 (11.2%)	0	4/143 (2.8%)
60	Malformations of cortical development: Fetal imaging and genetics	Wang, L et al, 2024	China	29	Central Nervous System – cortical malformations	Selected	ES Solo	19/29 (65.5%)	ND	ND
61	Diagnostic yield of genome sequencing for prenatal diagnosis of fetal structural abnormalities	Wang, Y et al, 2022	Canada	35	Major Fetal Abnormality not specified to one system	Unselected	GS Solo	5/35 (14.3%)	7/35 20.0%)	ND
62	Genetic diagnosis of fetal microcephaly at a single tertiary center in China	Wang, Y et al, 2023	China	162	Central Nervous System – microcephaly	Exploratory	ES Trio	37/162 (22.8%)	33/162 (20.4%)	ND
63	Prenatal genetic diagnosis associated with fetal ventricular septal defect: an assessment based on chromosomal microarray analysis and exome sequencing	Wang, Y et al, 2023	China	51	Cardiac – ventricular septal defect	Exploratory	ES Trio	6/51 (11.8%)	9/51 (17.6%)	1/51 (2.0%)
64	Chromosome Microarray Analysis and Exome Sequencing: Implementation in Prenatal Diagnosis of Fetuses with Digestive System Malformations	Wang, Y et al, 2023	China	143	Gastro-intestinal	Exploratory	ES Trio	23/143 (16.1%)	26/143 (18.2%)	ND
65	Detection of genomic variants by genome sequencing in foetuses with central nervous system abnormalities	Wang, Y et al, 2024	China	17	Central Nervous System	Selected	GS Trio	5/17 (29.4%)	4/17 (23.5%)	3/17 (17.6%)

66	Prenatal diagnosis of fetuses with ultrasound anomalies by whole-exome sequencing in Luoyang city, China	Wang, Y et al, 2024	China	48	Major Fetal Abnormality not specified to one system	Unselected	ES Solo	14/48 (29.2%)	15/48 (31.3%)	2/48 (4.2%)
67	Prenatal Diagnosed Agenesis of the Corpus Callosum: Identifying the Underlying Genetic Etiologies	Wei et al, 2024	China	26	Central Nervous System – ACC	Selected	ES Solo	12/26 (46.2%)	1/26 (3.8%)	ND
68	Exome sequencing in fetuses with congenital diaphragmatic hernia in a nationwide cohort	Weller et al, 2024	Nether-lands	68	Pulmonary – congenital diaphragmatic hernia	Exploratory	CES Trio (then 'open exome' if CES non-diagnostic	6/68 (8.8%)	ND	ND
69	Diagnostic yield using whole-genome sequencing and in-silico panel of 281 genes associated with non-immune hydrops fetalis in clinical setting	Westenius et al, 2022	Sweden	23	Hydrops	Selected	GS Solo with hydrops panel (281 genes)	12/23 (52.2%)	0	ND
70	Whole-genome sequencing in prenatally detected congenital malformations: prospective cohort study in clinical setting	Westenius et al, 2024	Sweden	50	Major Fetal Abnormality not specified to one system	Unselected	GS Trio with OMIM gene panel	13/50 (26.0%)	2/50 (4.0%)	1/50 (2.0%)
71	Genetic analysis of pregnancy loss and fetal structural anomalies by whole exome sequencing	Xiang et al, 2024	China	286	Major Fetal Abnormality not specified to one system	Unclear	ES Solo or Trio	74/286 (25.9%)	17/286 (5.9%)	ND
72	Prenatal diagnosis for fetuses with isolated and non-isolated congenital heart defects using chromosomal microarray and exome sequencing	Xing et al, 2022	China	47	Cardiac	Exploratory	CES Trio	7/47 (14.9%)	ND	ND
73	Prenatal genetic diagnosis of fetuses with dextrocardia using whole exome sequencing in a tertiary center	Xue, H et al, 2024	China	15	Cardiac – dextrocardia	Exploratory	ES Trio	3/15 (20.0%)	2/15 (13.3%)	5/15 (33.3%)

74	Prenatal diagnosis of fetal skeletal anomalies via whole-exome sequencing in a tertiary referral center	Xue, H et al, 2024	China	78	Skeletal	Selected	ES Trio	32/78 (41.0%)	9/78 (11.5%)	2/78 (2.6%)
75	Exome sequencing improves genetic diagnosis of congenital orofacial clefts	Yan et al, 2023	China	107	Craniofacial – cleft lip and palate	Exploratory	ES Trio	12/107 (11.2%)	8/107 (7.5%)	ND
76	Genomic architecture of fetal central nervous system anomalies using whole-genome sequencing	Yang et al, 2022	China	123	Central Nervous System	Unselected	GS Solo	27/123 (22.0%)	ND	ND
77	Exome sequencing as first-tier test for fetuses with severe central nervous system structural anomalies	Yaron et al, 2022	Israel	86	Central Nervous System	Selected	ES Trio	38/86 (44.2%)	9/86 (10.5%)	ND
78	Genetic aetiology distribution of 398 foetuses with congenital heart disease in the prenatal setting	Yi et al, 2023	China	301	Cardiac	Exploratory	ES Solo or Trio	32/301 (10.6%)	16/301 (5.3%)	ND
79	Genetic etiology of agenesis of the corpus callosum: a retrospective single-center cohort analysis of 114 fetuses	Yu, H et al, 2024	China	66	Central Nervous System - ACC	Selected	WES Trio	24/66 (36.4%)	6/66 (9.1%)	ND
80	Prenatal isolated clubfoot increases the risk for clinically significant exome sequencing results	Yu, Q et al, 2022	China	38	Skeletal – isolated talipes	Exploratory	ES Trio	4/38 (10.5%)	ND	ND
81	Exome Sequencing in Fetuses With Bilateral Renal Agenesis Identified on Second Trimester Ultrasound: A Single Referral Center Experience	Yu, Q et al, 2024	China	14	CAKUT – bilateral renal agenesis	Exploratory	ES Trio	6/14 (42.9%)	ND	ND
82	Simultaneous CNV-seq and WES: an effective strategy for molecular diagnosis of unexplained fetal structural anomalies	Zhang, H et al, 2024	China	159	Major Fetal Abnormality not specified to one system	Unselected	ES Solo	34/159 (21.4%)	ND	ND

83	Optimal prenatal genetic diagnostic approach for posterior fossa malformation: karyotyping, copy number variant testing, or whole-exome sequencing?	Zhang, J et al, 2024	China	28	Central Nervous System – posterior fossa malformation	Exploratory	ES Trio	9/28 (32.1%)	ND	ND
84	Investigation of the genetic and clinical features of laterality disorders in prenatal diagnosis: discovery of a novel compound heterozygous mutation in the DNAH11 gene	Zhang, S et al, 2024	China	16	Cardiac – laterality disorders	Exploratory	ES Trio	1/16 (6.3%)	1/16 (6.3%)	ND
85	Genetic correlation between fetal nuchal translucency thickening and cystic hygroma and exploration of pregnancy outcome	Zheng et al, 2024	China	35	Increased Nuchal Translucency	Exploratory	ES Trio	12/35 (34.3%)	ND	ND
86	Prenatal exome sequencing analysis in fetuses with central nervous system anomalies	Zhi et al, 2023	China	167	Central Nervous System	Selected	ES Solo or Trio	42/167 (25.1%)	16/167 (9.6%)	6/167 (3.6%)
87	Prenatal Diagnosis and Outcomes in Fetuses with Hemivertebra	Zhou, H et al, 2022	China	16	Skeletal – hemivertebrae	Exploratory	ES Trio	3/16 (18.8%)	3/16 (18.8%)	ND
88	Prenatal diagnosis and early childhood outcome of fetuses with extremely large nuchal translucency	Zhou, H et al, 2023	China	20	Increased Nuchal Translucency	Exploratory	CES Trio	2/20 (10.0%)	ND	ND
89	Utility of trio-based prenatal exome sequencing incorporating splice-site and mitochondrial genome assessment in pregnancies with fetal ultrasound anomalies: prospective cohort study	Zhu et al, 2022	China	90	Major Fetal Abnormality not specified to one system	Unselected	ES Trio	28/90 (31.1%)	6/90 (6.7%)	7/90 (7.8%)

Abbreviations: ACC: agenesis of corpus callosum, CAKUT: congenital abnormality of the kidneys and urinary tract, CES: clinical exome sequencing, CHD: congenital heart defects, ND: not described, SF: secondary findings, VUS: variants of uncertain significance, ES: exome sequencing, GS: genome sequencing.
